# Diagnostic sensitivity of carbohydrate deficient transferrin in heavy drinkers

**DOI:** 10.1186/1471-230X-14-97

**Published:** 2014-05-22

**Authors:** Kevin J Fagan, Katharine M Irvine, Brett C McWhinney, Linda M Fletcher, Leigh U Horsfall, Lambro Johnson, Peter O’Rourke, Jennifer Martin, Ian Scott, Carel J Pretorius, Jacobus PJ Ungerer, Elizabeth E Powell

**Affiliations:** 1 Department of Gastroenterology and Hepatology, Princess Alexandra Hospital, Woolloongabba 4102, Brisbane, Queensland, Australia; 2 Centre for Liver Disease Research, School of Medicine, The University of Queensland, Brisbane, Australia; 3 School of Medicine, The University of Queensland, Brisbane, Australia; 4 Pathology Queensland, Royal Brisbane and Women’s Hospital, Brisbane, Australia; 5 Cancer and Population Studies Group, Queensland Institute of Medical Research, Brisbane, Australia; 6 Division of Medicine, Princess Alexandra Hospital, Brisbane, Australia

**Keywords:** Alcohol, High performance liquid chromatography, Cirrhosis, Biomarker, Obesity

## Abstract

**Background and Aim:**

Carbohydrate deficient transferrin (CDT) is the most specific serum biomarker of heavy alcohol consumption, defined as ≥ 350–420 g alcohol/week. Despite introduction of a standardized reference measurement technique, widespread use of CDT remains limited due to low sensitivity. The aim of this study was to determine the factors that affect diagnostic sensitivity in patients with sustained heavy alcohol intake.

**Methods:**

Patients with a self-reported history of sustained heavy alcohol consumption were recruited from the hepatology outpatient department or medical wards. Each patient was interviewed with a validated structured questionnaire of alcohol consumption and CDT analysis using the standardized reference measurement technique with high performance liquid chromatography was performed on serum collected at time of interview.

**Results:**

52 patients were recruited: 19 from the hepatology outpatient department and 33 from general medical wards. Median alcohol intake was 1013 (range 366–5880) g/week over the preceding two week period. 26 patients had a diagnostic CDT based on a threshold value of %CDT > 1.7 indicating heavy alcohol consumption, yielding a sensitivity of 50%. Overweight/obesity (defined as body mass index (BMI) ≥ 25 kg/m^2^ in Caucasians and ≥ 23.0 kg/m^2^ in Asians), female gender and presence of cirrhosis were independently associated with non-diagnostic %CDT (≤ 1.7).

**Conclusions:**

CDT has limited sensitivity as a biomarker of heavy alcohol consumption. Caution should be applied when ordering and interpreting %CDT results, particularly in women, patients with cirrhosis and those with an elevated BMI.

## Background

The relative amount of serum carbohydrate-deficient transferrin (CDT) is currently the most specific serum biomarker of heavy alcohol consumption [1]. CDT refers to a temporary alteration in the glycosylation pattern of transferrin resulting in an increase in the relative amounts of disialo- and asialo-transferrin (and a decrease in tetrasialotransferrin) that occurs as a result of sustained heavy alcohol consumption (thresholds range from 50-80 g of alcohol/day for at least 2 weeks). Altered transferrin glycosylation patterns return to baseline levels within 2 to 5 weeks following complete abstinence from alcohol [2]. Using the standardized reference measurement technique with high performance liquid chromatography (HPLC) and quantification of disialotransferrin as a percentage of total transferrin (%CDT), a value of > 1.7 is considered to be specific for sustained heavy alcohol consumption [3]. Very few circumstances are associated with “false-positive” %CDT results using HPLC. These include genetic transferrin variants, [4] rare congenital disorders of glycosylation [5] and pregnancy [6,7].

In contrast to the high specificity, diagnostic sensitivity of %CDT for detection of heavy alcohol intake is low. Previous studies using older methods of CDT analysis such as immunoassays and anion-exchange methods have identified several patient characteristics that affect diagnostic sensitivity [8-13]. These characteristics include gender and metabolic risk factors such as obesity, insulin resistance, hypertension and dyslipidemia. We recently examined the diagnostic utility of %CDT in a hepatology outpatient setting [14]. Although few patients reported heavy alcohol consumption at the time of study, those acknowledged heavy drinkers with a body mass index (BMI) in the overweight or obese range had significantly lower %CDT values than lean heavy drinkers [14]. Neither the presence of compensated chronic liver disease, nor the etiology of non-alcoholic liver disease influenced interpretation of the CDT results. A key limitation of our earlier study and other previous studies investigating %CDT is the inclusion of patients with a broad range of alcohol intake and a relatively small proportion of patients with a heavy alcohol intake, at a level expected to cause %CDT > 1.7.

Despite recognition that clinical history and self-report screening tests are efficient methods to identify at-risk patients, there is clearly a need for an objective biomarker to support clinical suspicion of heavy alcohol intake. In order to improve the clinical utility of CDT measurements, factors that affect the diagnostic sensitivity and specificity need to be clearly defined, so that the test is requested and interpreted appropriately. The aim of this study was to determine in patients with *sustained heavy alcohol intake*, whether the level of %CDT is influenced by BMI or other clinical variables such as gender, age, ethnicity and smoking. To our knowledge, this is the first time that these factors have been examined in a cohort of patients with sustained heavy alcohol consumption.

## Materials and methods

### Patients and clinical data

Patients with self-reported heavy alcohol consumption were recruited from the hepatology outpatient department or medical wards at the Princess Alexandra Hospital, Brisbane, Australia during 2012 and 2013. Informed written consent was obtained from each patient and the protocol was approved by Metro-South-Health and the University of Queensland Human Research Ethics Committees. Those who agreed to participate were interviewed by the research co-ordinator using a structured questionnaire and a standard drink guide.

The questionnaire included an alcohol calendar to record alcohol consumption over the prior 4-week period and further direct questions to determine whether the calendar reflected usual alcohol consumption. It also recorded any previous periods of heavy alcohol consumption, defined as ≥ 350 g/week for females and ≥ 420 g/week for males for ≥ 6 months. These questions were supplemented by validated alcohol screening tools; the Alcohol Use Disorders Identification Test (AUDIT) [15] and the Brief Michigan Alcoholism Screening Test (BMAST) [16], to confirm current heavy alcohol consumption (as previously defined) and identify alcohol dependence.

Measurements of weight and height were obtained from patients at the time of interview. BMI was calculated as weight in kg/(height in meters)^2^. BMI was classified as lean (< 25 kg/m^2^ in Caucasians, < 23 kg/m^2^ in Asians), overweight (25–29.9 kg/m^2^ in Caucasians, 23.0 to 24.9 kg/m^2^ in Asians) or obese (≥ 30 kg/m^2^ in Caucasians, ≥ 25.0 kg/m^2^ in Asians). Lean body weight (LBW) was calculated using the Janmahasatian equation, as this has been validated in an obese population [17], and then used to estimate the volume of distribution (Vd) of alcohol, since fat has little water.

The medical record was reviewed to ascertain demographic details, previously diagnosed liver disease and other medical conditions, medications and history of alcohol, tobacco and recreational drug use. Standard biochemical and serological assays, liver imaging and histological assessment of a liver biopsy (if performed) were used to assess diagnosis and etiology of liver disease. In the absence of a liver biopsy, cirrhosis was determined on the basis of a Fibroscan® result > 14 kPa [18,19] and/or liver imaging (nodular or irregular liver surface and/or features of portal hypertension) in conjunction with other clinical and/or biochemical parameters. The severity of liver disease was evaluated using the Child-Turcotte-Pugh (CTP) classification. All patients with chronic hepatitis C had detection of circulating HCV RNA by polymerase chain reaction using the Abbott m2000 RealTime System (Abbott Laboratories, Illinois, USA). Routine haematological and biochemical tests were performed within 1–3 days of interview and serum collection for CDT analysis.

### CDT analysis

Serum was collected at the time of interview and stored at -80°C, a condition under which the transferrin isoform pattern is stable [20]. CDT analysis was performed on a Waters HPLC System (Waters Corporation Milford MA USA) as previously described [14]. The currently accepted laboratory reference value indicative of heavy drinking is %CDT > 1.7 [3].

### Statistical methods

Statistical analyses were performed in SPSS, employing Fisher’s exact test for categorical variables, either t-test or Mann–Whitney U-test for continuous variables and Spearman correlation analysis for univariate tests. Logistic regression with backward elimination of non-significant terms was used for multivariate models. A p-value of < 0.05 was considered statistically significant.

## Results

### Patient characteristics

Overall, 19 patients were recruited from the hepatology outpatient department and 33 were approached within 48 hours of admission to a general medical ward. All 52 patients reported previous periods of heavy alcohol consumption and excessive alcohol use during the 4 weeks prior to interview, with a median intake of 1013 (range 366–5880) g/week over the preceding 2 week period. In the general medicine group, the reason for presentation was: alcohol intoxication/withdrawal symptoms (n = 21), alcoholic hepatitis (n = 4), gastrointestinal bleed (n = 3), infection (n = 4) and pancreatitis (n = 1). Overall, the mean age of subjects was 50.3 (±11.8) years, 37 (71.2%) were men and 45 (86.5%) were Caucasian. BMI was lean in 27 patients (51.9%), overweight in 12 (23.1%), and obese in 13 (25%).

Eighteen patients (34.6%) had cirrhosis as determined by liver biopsy or imaging and 15 patients had evidence of concurrent hepatitis C infection (HCV). Other chronic medical conditions included: type 2 diabetes (n = 6), hypertension (n = 20), hyperlipidaemia (n = 9), rheumatoid arthritis (n = 2), COPD/asthma (n = 9), chronic kidney disease (CKD) > stage 3 (eGFR ≤ 59) (n = 2).

### Characteristics of patients with %CDT ≤ or > 1.7

Despite all 52 patients demonstrating heavy drinking based on results of questionnaires, only 26 had a %CDT > 1.7. The characteristics of patients with %CDT ≤ or > 1.7 are detailed in Table 1. A statistically significant difference in BMI was seen between heavy drinkers with a “diagnostic” or “non-diagnostic” %CDT. The mean (+/- SD) BMI of heavy drinkers with %CDT > 1.7 was 23.3 (+/- 3.9) kg/m^2^, with 73.1% within the lean weight range. In contrast, the mean (+/- SD) BMI for heavy drinkers with %CDT ≤ 1.7 was 28.2 (+/- 7.2) kg/m^2^, with only 30.8% within the lean weight range. Eighteen of 25 patients (72%) with BMI in the overweight/obese range had %CDT ≤ 1.7. The two overweight/obese patients with notably raised %CDT had CKD stage 3, with moderately reduced kidney function (eGFR 30–59). The presence of hypertension did not differ in relation to %CDT ≤ or > 1.7. Diabetes and hyperlipidemia were infrequent comorbidities in this group of patients and therefore their impact could not be evaluated.

**Table 1:** Demographic and clinical details of patients in relation to the %CDT reference cut-off value of 1.7

	**%CDT ≤ 1.7**	**%CDT > 1.7**	**P-value**
Subjects (n)	26	26	
Caucasian (n, %)	22 (84.6)	23 (88.5)	1.00
Age (years) mean (±SD)	51.1 (±10.2)	49.6 (±13.3)	0.67
Gender (n, % men)	13 (50.0)	24 (92.3)	0.002
BMI (kg/m^2^) mean (±SD)	28.2 (±7.2)	23.3 (±3.9)	0.003
Smoker (n, %)	14 (53.8)	20 (76.9)	0.14
Median alcohol consumption last 2 weeks (g/week) (range)	868 (366–2100)	1258 (510–5880)	0.005
Median estimated alcohol/Vd (g/week/kg) (range)	17.3 (6.7–42.2)	24.1 (7.4-82.5)	0.007
AUDIT mean (±SD)	27.6 (±7.2)	28.7 (±6.9)	0.57
BMAST mean (±SD)	17.6 (±8.1)	22.5 (±6.2)	0.018
Cirrhosis (n, %)	15 (57.7)	3 (11.5)	0.001

Fifteen of 18 patients (83.3%) with cirrhosis had a non-diagnostic %CDT. Of these 15 patients, 7 had compensated disease (CTP score A), 7 had functional compromise (CTP score B) and 1 had decompensated liver disease (CTP score C). The 3 cirrhotic subjects with %CDT > 1.7 had compensated disease (CTP score A). A statistically significant difference was also seen between gender and %CDT category, with women far less likely than men, to have a diagnostic %CDT. In contrast, ethnicity, age, and smoking status were comparable between the %CDT categories.

**Figure 1:**
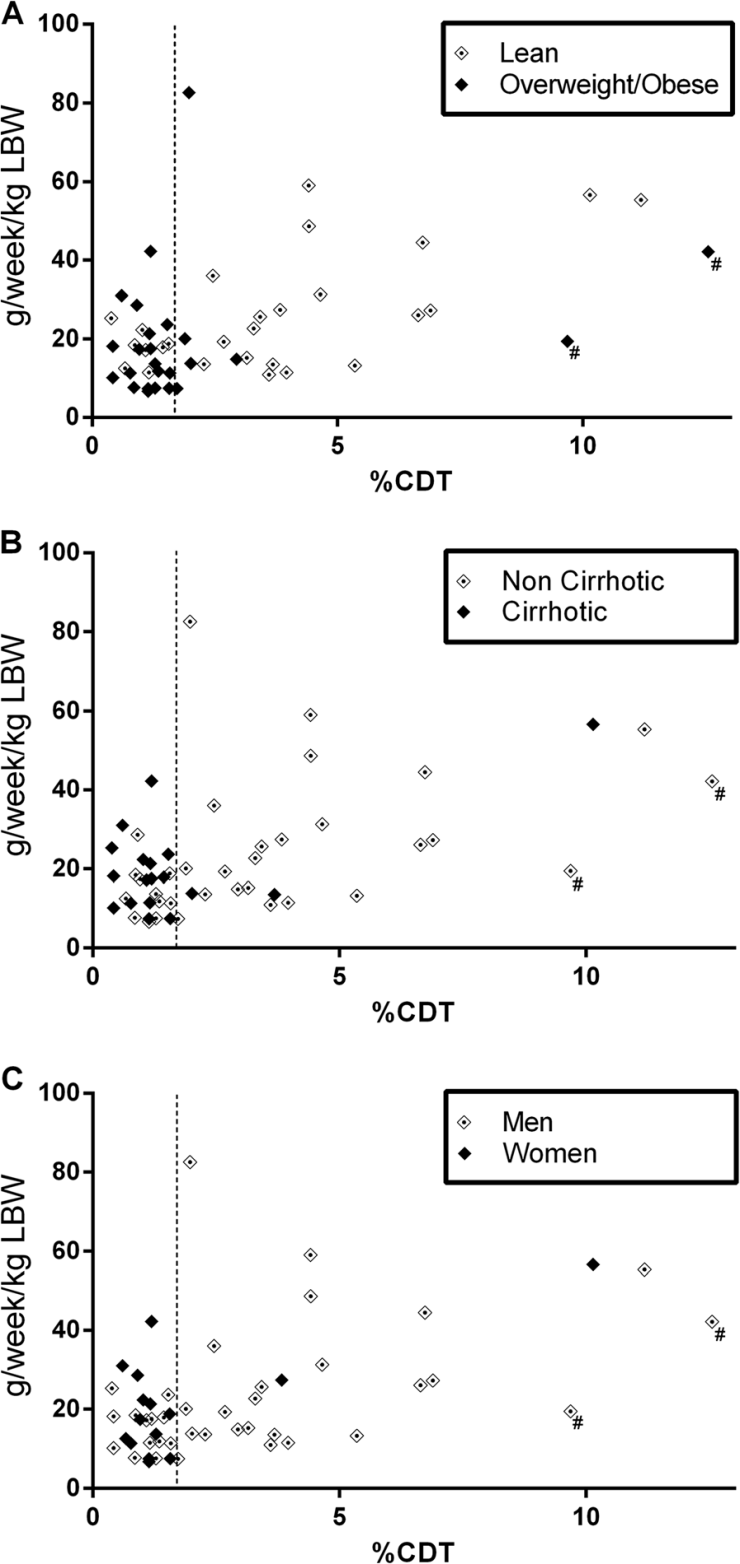
**Correlation of alcohol consumption (g/wk/kg LBW) and %CDT for: (A) lean vs. overweight/obese subjects; (B) non cirrhotic vs. cirrhotic subjects; and (C) men vs. women.** (^#^identifies the 2 patients with moderately decreased renal function (eGFR 30–59)). (LBW = Lean body weight). The vertical line represents 1.7% CDT.

Median alcohol consumption over the 2 weeks prior to interview was higher for patients with %CDT > 1.7 (1257.5 g/week) compared to subjects with %CDT ≤ 1.7 (867.5 g/week; p < 0.005). (Table 1) To consider the effects of body size and composition on alcohol concentrations, alcohol consumption was corrected for apparent volume of distribution (Vd) of alcohol using estimated lean body weight (LBW) as a surrogate for Vd. Median alcohol consumption per estimated Vd was 17.3 and 24.1g/week/kg LBW in patients with %CDT ≤ and > 1.7 respectively (P < 0.007). (Table 1) Alcohol consumption (g/wk/kg LBW) and %CDT were correlated, but the correlation was better for lean (r_s_ = 0.51, P < 0.01) than overweight subjects (r_s_ = 0.18, P = 0.40), non-cirrhotic (r_s_ = 0.54, P < 0.001) compared with cirrhotic subjects (r_s_ = 0.02, P = 0.94) and males (r_s_ = 0.48, P < 0.01) compared with females (r_s_ = 0.15, P = 0.60) (Figure 1).

Selected laboratory data of patients in relation to the %CDT reference cut-off value of 1.7 are detailed in Table 2. No statistically significant differences between the two groups for laboratory tests commonly used in clinical practice to suggest sustained heavy alcohol use (serum aminotransferases, gamma-glutamyltransferase, platelet count and mean corpuscular volume) were seen.

**Table 2:** Selected laboratory data of patients in relation to the %CDT reference cut-off value of 1.7

**Laboratory test, median (interquartile range)**	**Normal range**	**%CDT ≤ 1.7**	**%CDT >1.7**	**P-value**
Alkaline phosphatase (U/L)	*53-128*	101.5 (77.0-161.5)	90.5 (72.8-106.3)	0.16
Gammaglutamyl transferase (U/L)	*< 55*	182.0 (110.0-513.3)	135.5 (43.8-283.3)	0.09
Alanine transaminase (U/L)	*< 45*	54.0 (26.0-89.0)	57.5 (28.0-93.5)	0.98
Aspartate transaminase (U/L)	*< 35*	100.0 (49.0-163.5)	76.0 (47.0-139.8)	0.41
Platelets (× 10^9^/L)	*140-400*	165.5 (94.3-209.3)	168 (130.5-211.5)	0.37
Mean cell volume (fL)	*80-100*	98.5 (95.8-101.5)	96.5 (90.8-100.3)	0.16

Following multivariate analysis initially including age, gender, cirrhosis, BMI category, alcohol consumption and smoking status, overweight/obesity (OR = 5.8, p = 0.047), presence of cirrhosis (OR = 17.2, p = 0.007), female gender (OR = 14.3, p = 0.019) and lower alcohol consumption (OR = 0.998, p = 0.029) remained independently associated with %CDT ≤ 1.7. (Table 3).

**Table 3:** Variables independently associated with a non-diagnostic %CDT identified by logistic regression

		**95% Confidence intervals**		
**Variable**	**Odds ratio**	**Lower**	**Upper**	**P-value**
Gender (Women)	14.3	1.5	132.0	0.019
BMI (Overweight/Obese)	5.8	1.0	32.9	0.047
Cirrhosis (Yes)	17.2	2.2	137.0	0.007
Alcohol consumption over prior 2 weeks (g/week)	0.998	0.996	1.000	0.029

For categorical variables, the odds ratio refers to the category shown in brackets; for alcohol consumption the odds ratio refers to the decreased likelihood of a non-diagnostic %CDT per increase in alcohol consumption by 1 g/week.

## Discussion

Although %CDT (determined by the HPLC assay) remains the most specific serum biomarker of prolonged heavy alcohol consumption [1], its widespread use in clinical practice remains limited, largely due to concern about poor sensitivity and uncertainty about the factors that impact on CDT response to alcohol. This study was undertaken to identify clinical variables that affect the sensitivity of the standardized HPLC-based CDT assay in detecting heavy drinkers. Our study shows that only 50% of subjects drinking >50-60 g alcohol daily for at least 2 weeks had a %CDT > 1.7%, indicative of heavy alcohol intake. Overweight/obesity, the presence of cirrhosis and female gender were independently associated with a non-diagnostic %CDT level (≤ 1.7).

Previous population-based studies measuring CDT by ion-exchange chromatography and immunoassay found several patient characteristics, including gender, a high BMI and an insulin-resistant phenotype (high triglycerides and low HDL-cholesterol) were associated with reduced sensitivity of the CDT response to alcohol [8,10]. In contrast, more recent studies that quantified CDT using the standardized HPLC method did not find any clinically significant differences in CDT in relation to gender or BMI [21]. The authors concluded that the earlier findings were related to the analytical techniques used for measurement of CDT, and that adjustment of reference intervals in relation to gender or BMI was not required [3,21]. However, a major limitation of these studies was the low or unclear number with confirmed heavy drinking. In our study involving only confirmed heavy drinkers, elevated BMI and female gender clearly reduce the diagnostic sensitivity of %CDT using the standardized HPLC method.

Reporting CDT as relative amount of total transferrin concentration rather than an absolute value has improved sensitivity and specificity of the assay [22]. Introduction of this method was expected to negate many of the factors attributed to gender (e.g., pregnancy, oestrogens and anaemia), since they can cause variations in total transferrin concentrations. However, recent reports using %CDT have demonstrated that gender differences [23] and pregnancy-related changes in CDT isoform levels occur, although no biologic mechanism has been described [7,24]. Women may differ in the CDT isoforms that are increased by heavy alcohol intake, such as asialo- and monosialotransferrin, [25] neither of which are included in %CDT measurement using the new standardised HPLC technique. This would be in keeping with previous findings that women express higher CDT levels under basal conditions, but produce less in response to heavy drinking [26,27].

We previously investigated the diagnostic utility of %CDT in patients with liver disease, and found that heavy drinkers with a BMI in the overweight or obese range had significantly lower %CDT values than lean heavy drinkers [14]. The current study extends these findings by confirming the results in a larger group of subjects with confirmed heavy alcohol consumption and by showing that the effect of BMI is independent of other clinical variables. Interestingly 2 subjects had markedly elevated %CDT values (9.68% and 12.55%) despite overweight/obesity, in the setting of moderately decreased renal function (eGFR 30–59). Currently little is known regarding the process and elimination kinetics of CDT from the circulation and thus the mechanisms responsible for this effect are unclear, but may relate to altered elimination in the presence of renal failure [28]. Chronic kidney disease does not appear to cause an increase in the baseline levels of CDT in subjects without hazardous drinking [29]. Similarly, non-enzymatic glycation of transferrin, a process that may occur in uremia [28] and diabetic subjects [30] does not appear to interfere with HPLC-based CDT measurement [31].

In our prior study we found that the presence of cirrhosis due to various chronic liver diseases did not lead to “false positive” %CDT results [14]. In the current study of heavy drinkers, cirrhosis was associated with *reduced* sensitivity of the %CDT response to alcohol, which is contrary to some previous reports [32-34]. This finding confirms earlier studies using non-HPLC methods that found patients with cirrhosis and a high current alcohol intake had lower CDT values compared with “control” subjects without liver disease but drinking more than 50 g alcohol/day [35]. The reasons underlying these findings remain unclear. Transferrin is synthesised, glycosylated and secreted by the liver and the rate of transferrin synthesis is reduced in cirrhotic patients [36]. Furthermore insulin resistance is present in nearly all patients with cirrhosis [37] and thus similar mechanisms may reduce the CDT response to alcohol in the setting of cirrhosis and overweight/obesity.

## Conclusions

In conclusion, %CDT has limited sensitivity as an objective biomarker to identify subjects consuming harmful amounts of alcohol. In our cohort of sustained heavy drinkers, diagnostic sensitivity of %CDT was 50% and yielded false negative results in particular patient subgroups: women, patients with cirrhosis and those with an elevated BMI. Therefore caution should be applied when ordering and interpreting %CDT results in these subject populations. Further studies with larger numbers of well-characterised patients, who consume heavy amounts of alcohol, are required to further assess factors which impact on the sensitivity of this assay.

## Competing interests

The authors declare that they have no competing interests.

## Authors’ contributions

KF conceived and coordinated the study, collected patient data and blood samples, contributed to analysis of data and wrote the manuscript. KI contributed to analysis of data and writing the manuscript. LH collected patient data and blood samples and contributed to the manuscript. BM, LJ, JU, CP performed the CDT analysis and contributed to data analysis and writing the manuscript. PO performed the statistical analysis and contributed to the manuscript. LF, JM and IS contributed to data analysis and writing the manuscript. EP conceived the study and contributed to analysis of data and writing the manuscript. All authors read and approved the final manuscript.

## Pre-publication history

The pre-publication history for this paper can be accessed here:

http://www.biomedcentral.com/1471-230X/14/97/prepub
